# Mathematical modeling of flexible printed circuit configuration: a study in deformation and optimization

**DOI:** 10.1038/s41598-024-64770-6

**Published:** 2024-06-19

**Authors:** Longhui Meng, Liang Ding, Aqib Mashood Khan, Mohammed Alkahtani, Yicai Shan

**Affiliations:** 1 School of Mechanical and Power Engineering, Nanjing Tech University, Nanjing, 211816 China; 2 Nanjing WIT Science and Technology Co., Ltd, Nanjing, 210012 China; 3 College of Mechanical and Electrical Engineering, Nanjing University of Aeronautics and Astronautics, Nanjing, 210016 China; 4 Department of Industrial Engineering, College of Engineering, King Saud University, P.O. Box 800, 11421 Riyadh, Saudi Arabia; 5 School of Electronic Engineering, Nanjing Xiaozhuang University, Nanjing, 211171 China

**Keywords:** Flexible printed circuits (FPCs), Finite element method (FEM), Curvature optimization, Jacobi matrix iterative method, Electronic circuit design, Applied mathematics, Computational science

## Abstract

This manuscript offers an exhaustive analysis of Flexible Printed Circuits (FPCs), concentrating on enhancing their design to surmount two primary challenges. Firstly, it seeks to obviate contact with proximate components. Secondly, it aspires to adhere to pre-established curvature constraints. Predicated on the curvature properties of FPCs, we have developed a model adept at accurately forecasting FPC deformation under diverse conditions. Our inquiry entails a thorough examination of various FPC configurations, including bell, 'U', and 'S' shapes. Central to our methodology is the strategic optimization of FPC spatial arrangements, aiming to avert mechanical interference and control curvature, thus mitigating mechanical strain. This dual-faceted strategy is pivotal in enhancing the durability and operational reliability of FPCs, particularly in contexts demanding elevated flexibility and precision. Our research offers essential insights into the refinement of FPC design, skillfully addressing the complexities associated with curvature and physical interaction. Collectively, this study advocates a comprehensive framework for the design and implementation of FPCs, significantly advancing the field of contemporary electronics by ensuring these components meet the evolving demands of the industry.

## Introduction

**Figure 1:**
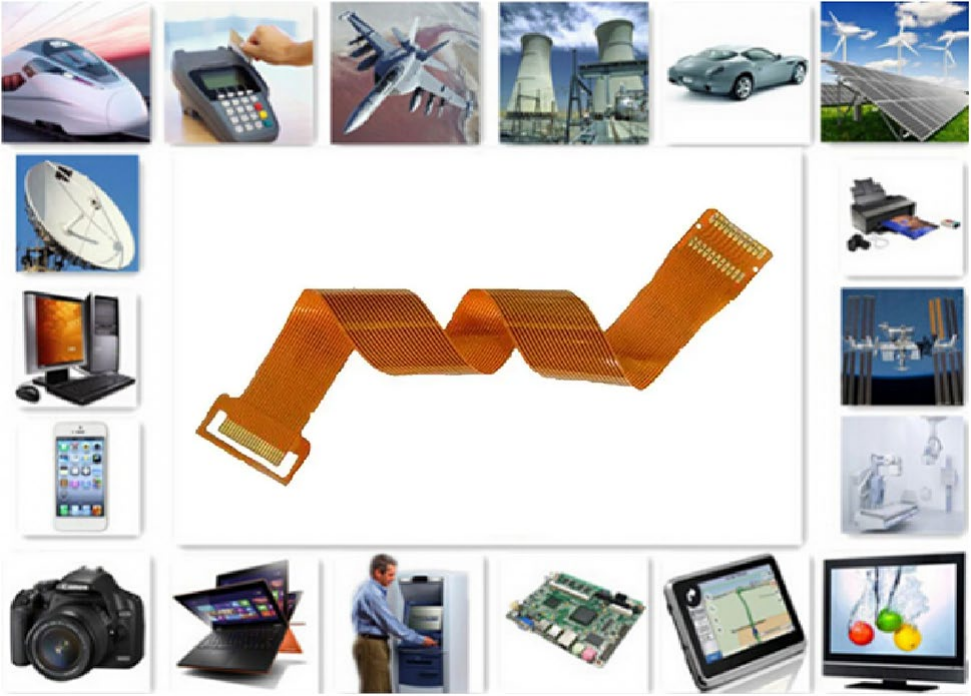
Versatile Applications of Flexible Printed Circuits (FPC) in Modern Technology^44^.

In contemporary electronics, Flexible Printed Circuits (FPCs) have become pivotal, transforming electronic device design and function^1–3^. These circuits surpassing traditional rigid Flexible Printed Circuit Boards (PCBs), offer unmatched flexibility and adaptability due to their construction from pliable, high-quality materials like polyimide. Their ability to fold or twist into small or irregular spaces enhances durability under physical stress. FPCs are especially beneficial for their space and weight efficiency, consolidating multiple rigid boards and wiring into lighter, more compact assemblies^4–6^. This is crucial in sectors like aerospace, wearable technology, and mobile devices, where reducing space and weight is essential. Additionally, their inherent flexibility allows them to be used in dynamic applications without sacrificing circuit performance or integrity. Their applications span various industries, which is as shown in Fig. 1^7^. In the medical field, FPCs contribute to compact, flexible devices like sensors and implants, improving patient comfort and effectiveness^8–11^. The automotive industry uses them in systems like dashboard displays and sensor networks, valuing their resistance to vibrations and temperature changes. In consumer electronics, FPCs are key to the thinness of smartphones, tablets, and laptops^12–15^. Moreover, they are central to the emerging field of flexible and wearable electronics, enabling innovative devices that align with ergonomic designs and human movement^16–20^.

In the field of electronic circuit design, particular attention must be paid to the use and placement of Flexible Printed Circuits. These components, known for their flexibility and adaptability in various applications, require careful handling and strategic positioning to ensure optimal performance and longevity^21^. The reasons for this careful approach are multifaceted:

In the design of Flexible Printed Circuits (FPCs), it is critical to address several considerations to ensure their durability and functionality. Firstly, FPCs are inherently more delicate than their rigid counterparts and are prone to mechanical damage, such as cracks or fractures^22^, particularly when subjected to external stress or forces^23^. This vulnerability is heightened in environments where the FPCs may contact other components, especially in settings prone to movement or vibration. A strategic physical separation can significantly reduce the risk of such damage.

Additionally, FPCs face the challenge of electrical interference when placed too close to other electronic components, particularly those generating strong electromagnetic fields^24^. This interference can disrupt the normal operation of the circuits on the FPC, leading to malfunctions or inefficiency. Furthermore, maintaining signal integrity is paramount^25^, as close proximity to other components can induce signal loss or interference, which is critical in high-frequency or precision applications where signal clarity and integrity are vital.

Effective thermal management is another key consideration^26,27^. If FPCs are positioned too close to heat-emitting components, the resultant high temperatures can adversely affect their performance and lifespan. Adequate spacing is essential to facilitate proper heat dissipation and prevent thermal overload, which can degrade the materials and circuits of the FPC. Finally, the unique flexibility of FPCs allows them to be bent or curved to fit specific designs, but this bending should be carefully controlled^28^. Excessive curvature can impose undue stress on the material, increasing the risk of fatigue and damage over time^29^. Proper management of curvature is essential to maintain the structural integrity of the FPC.

In summary, the strategic placement and handling of FPCs are crucial in circuit design. This involves not only avoiding physical contact with other components but also considering aspects like electromagnetic compatibility, signal integrity^30^, thermal management^31^, and mechanical stress^32–34^. These considerations are integral to the design process, ensuring that FPCs function effectively and reliably in their intended applications. This detailed understanding of the nuances associated with FPCs is vital for professionals in the field of electronic design and is worthy of thorough discussion in academic and technical literature.

In the field of FPC mechanics and deformation, John et al.^35^ delved into the mechanical and material challenges associated with bending, forming, and flexing printed circuits, specifically targeting non-glass woven materials. Their research provided a comprehensive analysis of the strain effects on copper layers during bending processes and its implications for both static and dynamic applications, underscoring the importance of understanding the material properties and copper's behavior under mechanical stress. Concurrently, Hirotaka et al.^36^ examined the performance characteristics of an FPC-based Planar Inverted F Antenna (PIFA), focusing on its robustness in maintaining stable radiation patterns and efficiency when subjected to mechanical deformations. Their findings confirmed the antenna's adaptability for use in compact mobile devices, with minimal performance degradation. Additionally, Ping et al.^22^ identified prevalent failure modes in FPC applications, such as via interconnection issues, trace cracks, and the black pad phenomenon. They proposed methods to enhance the reliability and quality of FPCs through improvements in manufacturing processes, mechanical design, and the distribution of dynamic stresses. Collectively, these studies illuminate the significant progress and persisting challenges in the design and application of FPCs within the realm of advanced electronics.

Building upon these foundational insights into FPC mechanics and deformations, further investigations have specifically targeted the complexities of Flexible Printed Circuit Boards (FPCBs). Recent studies have made significant contributions to this area. Chong et al.^37^ delved into the heat transfer and deformation aspects under thermal conditions, using fluid–structure interaction for simulations. Leong et al.^38^ aimed to optimize FPCBs to mitigate stress and deflection, employing advanced methodologies. Alexander^39^ concentrated on the mechanical reliability of FPCs, particularly in dynamic settings, integrating fatigue testing with computational analysis. Liu et al.^40^ examined the factors influencing FPC bending, revealing critical insights into material and design choices. Zhang et al.^41^ explored the reliability of rigid-flex PCBs under high acceleration, shedding light on stress dynamics. Lall et al.^42^ investigated deformation challenges during the soldering process, while Muhammad et al.^43^ assessed the post-soldering reliability of FPCBs, emphasizing manufacturing intricacies. Together, these studies significantly advance the understanding and design of FPCBs in microelectronics.

This research addresses a crucial gap in understanding and applying deformations of FPCs. We have extensively analyzed FPC curvature under varying conditions, an area previously understudied. Our methodology combines Finite Element Method (FEM) simulation using Abaqus 2020 for deformation behavior analysis and advanced mathematical modeling to examine curvature and spatial separation along the FPC.

In our study, we employed the Jacobi Matrix Iterative Method, significantly improving the precision of predicting FPC curvature. Our findings hold substantial importance for the design and manufacturing of FPCs. They highlight the necessity of maintaining a free contact space between FPCs and adjacent components, as well as optimizing curvature. This approach is crucial to prevent issues such as mechanical stress, inefficient heat dissipation, and physical damage, all of which can adversely affect circuit functionality and overall device reliability. Additionally, our research identified the ideal length for FPCs that minimizes maximum curvature, thereby achieving a balance between flexibility, structural integrity, and reliability.

In summary, our research marks a significant advancement in FPC study, offering new insights and methodologies. It aims to improve FPC behavior understanding under various conditions and lay a foundation for future FPC design and application enhancements, contributing significantly to device longevity and performance in the electronic design and manufacturing sector.

## Correlation between curvature and surface bending stress in FPC

Comprehending the deformation behavior of FPCs holds paramount importance, particularly concerning their curvature in response to diverse conditions. A critical component of this comprehension entails scrutinizing the interplay between the curvature of the FPC and the consequent bending stress it incurs.

**Figure 2:**
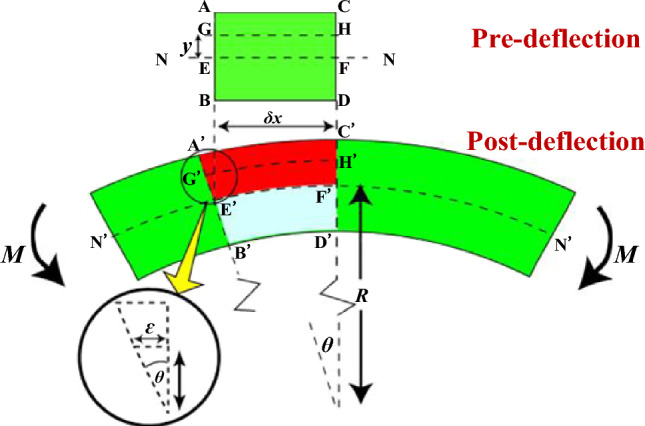
Comparative Analysis of FPC Deformation Pre- and Post-Deflection.

In Fig. 2, the FPC is depicted in its pre- and post-deflection states. Initially, the unbent state is marked by a linear neutral layer (N–N), where the materials of the FPC remain undeformed. Subsequently, in the post-deflection state, distinguished by a curved neutral layer (N'-N'), the segment E'F' (corresponding to the pre-bending line EF) within the neutral layer retains its length *δx*. As a result, in the bent configuration, the length of segment E'F' is equivalent to the product of radius R and angle *θ*.

Conversely, the segment GH in the pre-deflection state, which aligns with the arc G'H' in the post-deflection state, extends from *δx* to (*R* + *y*)·*θ* following the bending. The alteration in length, denoted as Δ*l*, is expressed as follows:

1 $$\Delta l = l_{{}} - l_{GH} = \left( {R + y} \right)\theta - R\theta = y\theta$$

Presuming the absence of plastic deformation, it is feasible to compute the elastic strain, *ε*, within the material layer situated a '*y*' distance from the neutral layer:

2 $$\varepsilon = \frac{\Delta l}{L} = \frac{y\theta }{{R\theta }} = \frac{y}{R}$$

The bending Stress, *σ*, is subsequently derived as follows:

3 $$\sigma = E\varepsilon = \frac{Ey}{R}$$

In this context, *E* denotes the Young’s modulus, epitomizing the inherent elasticity of the material. This formulation elucidates the direct correlation among bending stress *σ*, Young’s modulus *E*, strain *ε*, radial distance *y*, and curvature radius *R*.

In our analysis, we consider the flexible printed circuit (FPC) to have a uniform thickness denoted by '*h*'. This assumption allows us to treat the material as homogeneous across its thickness, simplifying our approach to evaluating its properties. Furthermore, we assume that the distance from any point on the surface to the neutral layer is half of the thickness, or '*h*/2'. This assumption is crucial for simplifying the computations of mechanical properties. Additionally, we presume that the FPC maintains a constant elastic modulus '*E*', which ensures consistent mechanical behavior throughout the analysis. These assumptions are foundational in facilitating a streamlined and effective evaluation of the FPC's performance under various conditions.

These presumptions facilitate a mathematical simplification in our model, culminating in a direct correlation between bending stress (*σ*) and curvature (1/*R*), as delineated in Eq. (4).

4 $$\sigma \propto \frac{1}{R}$$

This relationship accentuates the crucial nexus between FPC curvature and the distribution of surface bending stress.

## Finite element simulation and stress analysis

To elucidate the relationship between FPC curvature and bending stress, we constructed a finite element model (FEM) utilizing Abaqus 2020. The model's dimensions comprise a length of 60 mm, a width of 2 mm, and a thickness of 0.1 mm. We opted for a global element size of 0.2, stratifying the thickness direction into 10 elements to enhance resolution. The selected C3D8R element type, an 8-node linear brick with reduced integration and hourglass control, is renowned for its efficiency in modeling substantial deformations and displacements. The simulation employed a Static, General step with nonlinear geometry (Nlgeom) activated^45^, and the ultimate model configuration employed 30,000 elements to ensure a comprehensive analysis of the FPC.

**Figure 3:**
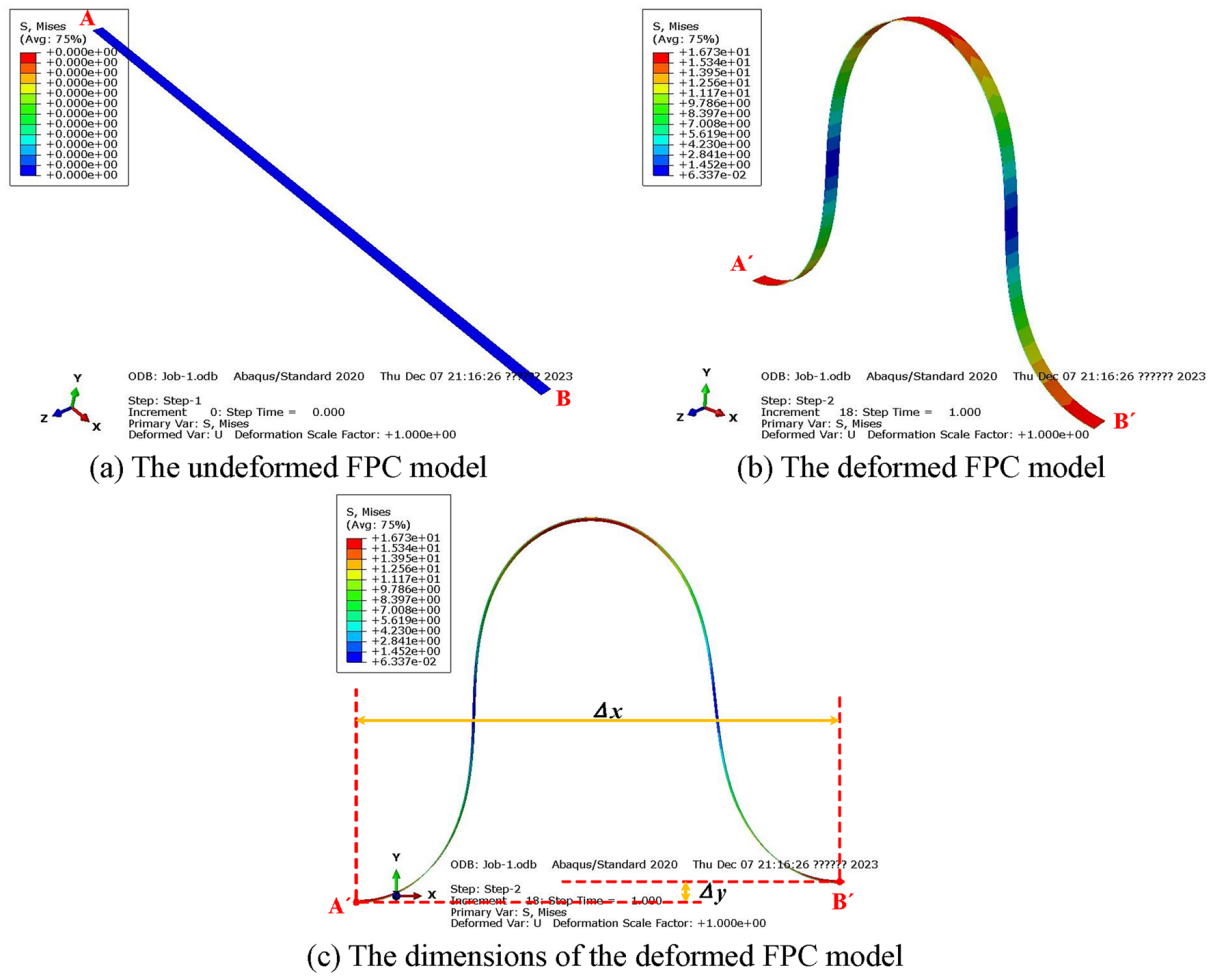
Finite Element Model of FPC and Its Dimensional Schematic.

In its nascent, unbent state, as illustrated in Fig. 3a, the FPC model displays a linear configuration. At the A end, a displacement of (25 mm, 0) alongside a rotation angle of 90° was imposed. Concurrently, the B end was subjected to a displacement of (25 mm, − 5 mm) coupled with a rotation angle of -90°.

The ensuing post-bending state of the FPC, manifesting as a bell shape, is depicted in Fig. 3b. This form elucidates the varying internal stresses and the coordinate discrepancies (Δ*x* and Δ*y*) between points A' and B', as expounded in Fig. 3c.

**Figure 4:**
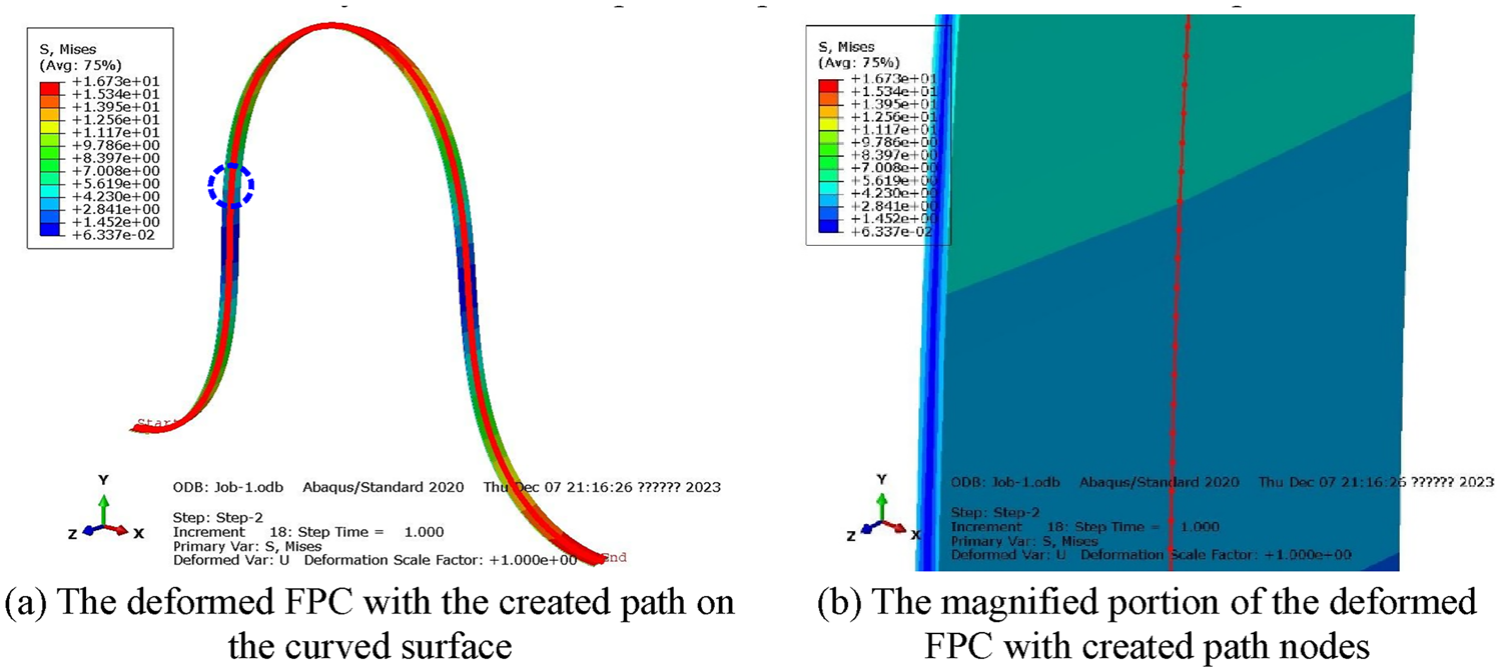
The Path Traced Along the Curved Surface of the FPC Model.

To precisely monitor the pattern of surface bending stress, a trajectory was marked along the curved surface of the FPC. This entailed creating a series of points, as detailed in Fig. 4a. A magnified depiction of the region within the blue circle from Fig. 4a is exhibited in Fig. 4b. This detailed view distinctly reveals that the path comprises a succession of discrete points.

**Figure 5:**
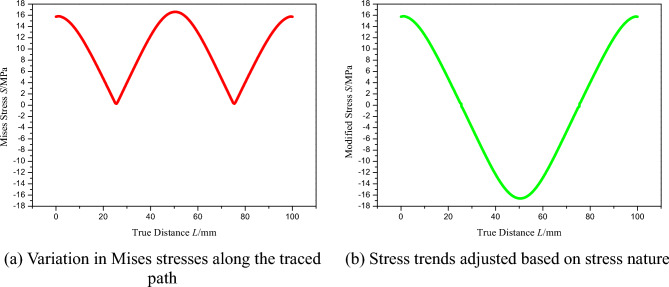
Analysis of Stress Variations and Adjustments in Traced Path.

The Von Mises stress profile, derived from the computational model and traced along the FPC's trajectory, is graphically represented in Fig. 5a. The calculation of Von Mises stress adheres to the established formula^46^:

5 $$\sigma_{{\text{Von Mises}}} = \sqrt {\left( {\sigma_{1} - \sigma {}_{2}} \right)^{2} + \left( {\sigma_{2} - \sigma_{3} } \right)^{2} + \left( {\sigma_{3} - \sigma_{1} } \right)^{2} + 6\left( {\tau_{1}^{2} + \tau_{2}^{2} + \tau_{3}^{2} } \right)}$$

Within this framework, *σ*₁, *σ*₂, and *σ*₃ denote the principal stresses from three-dimensional stress analysis, while *τ*₁, *τ*₂, *τ*₃ correspond to the related shear stresses. This equation is instrumental in ascertaining the likelihood of material yield or failure under multifaceted loading conditions.

Significantly, although Von Mises stress values are customarily non-negative, we have modified this approach to render a more exact portrayal of the FPC's curvature effects. Specifically, we regard compressive stresses as negative and tensile stresses as positive. This adjustment facilitates a more precise illustration of the stress profile attributed to curvature, as exemplified in Fig. 5b.

## Mathematical modeling and analysis of the free bending curve of FPC

### Mathematical modeling

**Figure 6:**
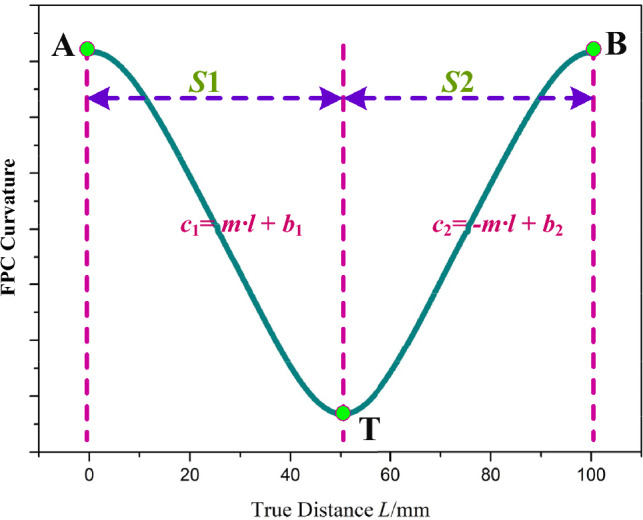
Relationship Between True Distance and Curvature of Deformed FPC.

Empirical examination of the data featured in Fig. 5b suggests that the trend of FPC curvature can be systematically partitioned into two discrete segments, labeled as *S*1 and *S*2. This bifurcation is explicitly delineated in Fig. 6.

Within each segment (*S*1 and *S*2), a linear relationship is discernible between the curvature of the FPC and its length. In Segment *S*1, the curvature-length relationship, with curvature represented as *c*_1_ and length as *l*, is articulated as:

6 $$c_{1} = ml + b_{1}$$

Herein, *c*_1_ signifies the curvature in Segment *S*1, m embodies the slope of the linear correlation, *l* denotes the length along the FPC, and *b*_1_ represents the intercept for *c*_1_.

In Segment *S*2, a similar yet inverse relationship is observed, denoted by:

7 $$c_{2} = - ml + b_{2}$$

where *c*_2_ represents the curvature in Segment *S*2, with ${b}_{2}$ denoting its intercept. The coordinates of the intersection point T, where *c*_1_ and *c*_2_ intersect, are determined as $\left(\frac{{b}_{2}-{b}_{1}}{2m},\frac{{b}_{1}+{b}_{2}}{2}\right)$.

To forecast the deformation curve of the FPC using the coordinates of points A and B, and the total length of the FPC, three pivotal parameters are necessitated: the slope '*m*' and the intercepts '${b}_{1}$' and '${b}_{2}$'. The ascertainment of these parameters entails a calculus-based examination of the correlation between curvature and spatial separation along the FPC, specifically concentrating on the distinct segments previously identified.

The differential increments in the X and Y directions along the FPC are mathematically delineated as:

8 $$dx = dl\cos \theta$$

9 $$dy = dl\sin \theta$$

**Figure 7:**
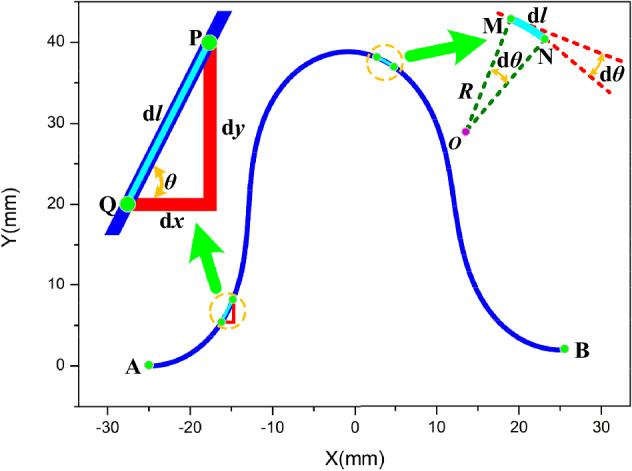
Analysis of the deformed FPC based on calculus principles.

In these equations, '*dl*' denotes the infinitesimal length increments along the FPC. '*θ*' is the tangent angle at any given point on the FPC curve, as depicted in Fig. 7.

It is pertinent to observe that the tangent angle '*θ*' fluctuates as a function of the distance '*l*' along the FPC curve from the initial point to a specified point. As a result, the displacements along the X and Y axes between points A' and B' can be computed employing integral calculus:

10 $$\Delta x = \int_{0}^{L} {\cos \left( {\theta \left( l \right)} \right)} dl$$

11 $$\Delta y = \int_{0}^{L} {\sin \left( {\theta \left( l \right)} \right)} dl$$

As portrayed in Fig. 6, understanding the relationship between the curvature of the FPC and its length necessitates addressing the variability of curvature within different segments. This demands an in-depth investigation of the function *θ*(*l*) and its alterations across these segments. Distinguishing these differences is essential to precisely delineate the subtle shifts in curvature along the FPC's trajectory.

For Segment *S*1, the differential increment of the tangent angle, denoted as $d{\theta }_{S1}$, is expressed as:

12 $$d\theta_{S1} = - c_{1} dl$$

The tangent angle formed by the tangent line at any given point on Segment *S*1 relative to the X-axis is designated as:

13 $$\theta_{S1} \left( l \right) = - \int_{0}^{l} {c_{1} dl} = - \int_{0}^{l} {\left( {ml + b_{1} } \right)dl}$$

The differential increments of the dimensions *dx*1 and *dy*1 in the X and Y directions, respectively, within Segment *S*1, can be mathematically articulated as follows:

14 $$dx1 = \cos \left( {\theta_{S1} \left( l \right)} \right)dl$$

15 $$dy1 = \sin \left( {\theta_{S1} \left( l \right)} \right)dl$$

Expanding on these interrelations, the variations in coordinates at the ends of the FPC within Segment *S*1, denoted as Δ*x*1 and Δ*y*1, can be mathematically formulated as follows:

16 $$\Delta x1 = \int_{0}^{{\frac{{b_{2} - b_{1} }}{2m}}} {\cos \left( {\theta_{S1} \left( l \right)} \right)} dl$$

17 $$\Delta y1 = \int_{0}^{{\frac{{b_{2} - b_{1} }}{2m}}} {\sin \left( {\theta_{S1} \left( l \right)} \right)} dl$$

For point T, as depicted in Fig. 6, which signifies the conclusion of Segment *S*1, the corresponding tangent angle, ${\theta }_{T}$, can be articulated as:

18 $$\theta_{{\text{T}}} = - \int_{0}^{{\frac{{b_{2} - b_{1} }}{2m}}} {\left( {ml + b_{1} } \right)} dl$$

Transitioning to Segment *S*2, the differential increment of the tangent angle can be mathematically delineated as follows:

19 $$d\theta_{S2} = - dlc_{2} = - dl\left( { - ml + mL/2} \right)$$

The tangent angle corresponding to any point situated within Segment *S*2 can be expressed as follows:

20 $$\theta_{S2} \left( l \right) = \theta_{{\text{T}}} - \int_{{\frac{{b_{2} - b_{1} }}{2 \cdot m}}}^{l} {c_{2} dl} = \theta_{{\text{T}}} - \int_{{\frac{{b_{2} - b_{1} }}{2 \cdot m}}}^{l} {\left( { - ml + b_{2} } \right)dl}$$

Concerning the differential increments of its dimensions, specifically *dx*2 and *dy*2, in the X and Y directions within Segment *S*2, these can be depicted as follows:

21 $$dx2 = \cos \left( {\theta_{S2} \left( l \right)} \right)dl$$

22 $$dy2 = \sin \left( {\theta_{S2} \left( l \right)} \right)dl$$

Elaborating on these interrelationships, the variations in coordinates at the termini of the FPC within Segment *S*2, represented by Δ*x*2 and Δ*y*2, can be mathematically represented as follows:

23 $$\Delta x2 = \int_{{\frac{{b_{2} - b_{1} }}{2m}}}^{L} {\cos \left( {\theta_{S2} \left( l \right)} \right)} dl$$

24 $$\Delta y2 = \int_{{\frac{{b_{2} - b_{1} }}{2m}}}^{L} {\sin \left( {\theta_{S2} \left( l \right)} \right)} dl$$

The cumulative dimensions of the FPC curve in both the X and Y directions may be formulated as follows:

25 $$\Delta x = \Delta x1 + \Delta x2 = \int_{0}^{{\frac{{b_{2} - b_{1} }}{2m}}} {\cos \left( {\theta_{S1} \left( l \right)} \right)} dl + \int_{{\frac{{b_{2} - b_{1} }}{2 \cdot m}}}^{L} {\cos \left( {\theta_{S2} \left( l \right)} \right)} dl$$

26 $$\Delta y = \Delta y1 + \Delta y2 = \int_{0}^{{\frac{{b_{2} - b_{1} }}{2m}}} {\sin \left( {\theta_{S1} \left( l \right)} \right)} dl + \int_{{\frac{{b_{2} - b_{1} }}{2m}}}^{L} {\sin \left( {\theta_{S2} \left( l \right)} \right)} dl$$

In this context, ${\theta }_{S1}$ and ${\theta }_{S2}$ are as explicated in Eqs. (14) and (21) respectively. Δ*x* and Δ*y* represent the differential coordinates at the termini of the FPC along the X and Y axes, respectively. '*L*' signifies the total length of the FPC curve, while '* b*_1_', '* b*_2_', and '*m*' pertain to the intercept and slope coefficients, as previously delineated.

For the tangent angle at the starting point A' of the post-bending curve, as illustrated In Fig. 3, it can be expressed as follows:

27 $$\theta_{A^{\prime}} = 0$$

While the corresponding tangent angle at the endpoint B' is

28 $$\theta_{B^{\prime}} = \theta_{{\text{T}}} - \int_{{\frac{{b_{2} - b_{1} }}{2m}}}^{L} {\left( { - ml + b_{2} } \right)dl} = 0$$

The disparity in the tangent angles at the starting point A' and the endpoint B' can be mathematically formulated as:

29 $$\Delta \theta = \theta_{B^{\prime}} - \theta_{A^{\prime}} = \theta_{{\text{T}}} - \int_{{\frac{{b_{2} - b_{1} }}{2m}}}^{L} {\left( { - ml + b_{2} } \right)dl - {\pi \mathord{\left/ {\vphantom {\pi 2}} \right. \kern-0pt} 2}} = 0$$

Equations (25), (26), and (29) underscore the need for three as-yet-unidentified variables, explicitly labeled as '*m*', '*b*_1_', and '*b*_2_'. Resolving these variables, which requires the application of a non-linear equation system approach, is crucial for precisely forecasting the curvature exhibited by the FPC. By utilizing Eqs. (13), (18), and (20), Eqs. (25), (26), and (29) can be reconstituted into a corresponding expression as follows:

30 $$F\left( u \right) = \left[ \begin{gathered} F1 \hfill \\ F2 \hfill \\ F3 \hfill \\ \end{gathered} \right] = \left[ \begin{gathered} \Delta x - \left[ {\int_{0}^{{\frac{{b_{2} - b_{1} }}{2m}}} {\cos \left( { - \int_{0}^{l} {\left( {ml + b_{1} } \right)dl} } \right)dl + \int_{{\frac{{b_{2} - b_{1} }}{2m}}}^{L} {\cos \left( { - \int_{0}^{{\frac{{b_{2} - b_{1} }}{2m}}} {\left( {ml + b_{1} } \right)dl - \int_{{\frac{{b_{2} - b_{1} }}{2m}}}^{l} {\left( { - ml + b_{2} } \right)dl} } } \right)dl} } } \right] \hfill \\ \Delta y - \left[ {\int_{0}^{{\frac{{b_{2} - b_{1} }}{2m}}} {\sin \left( { - \int_{0}^{l} {\left( {ml + b_{1} } \right)dl} } \right)dl + \int_{{\frac{{b_{2} - b_{1} }}{2m}}}^{L} {\sin \left( { - \int_{0}^{{\frac{{b_{2} - b_{1} }}{2m}}} {\left( {ml + b_{1} } \right)dl - \int_{{\frac{{b_{2} - b_{1} }}{2m}}}^{l} {\left( { - ml + b_{2} } \right)dl} } } \right)dl} } } \right] \hfill \\ \Delta \theta - \left[ { - \int_{0}^{{\frac{{b_{2} - b_{1} }}{2m}}} {\left( {ml + b_{1} } \right)dl - \int_{{\frac{{b_{2} - b_{1} }}{2 \cdot m}}}^{L} {\left( { - ml + b_{2} } \right)dl} } } \right] \hfill \\ \end{gathered} \right] = 0$$

In this context, *F*(*u*) is defined as a vector function, where $u=[m,{b}_{1},{b}_{2}]$, comprised solely of the three indeterminate variables: '*m*', '*b*_1_', and '*b*_2_'. Accurately ascertaining the values of these variables is essential for precisely delineating the curvature of the bent FPC.

### Nonlinear equation system solution

**Figure 8:**
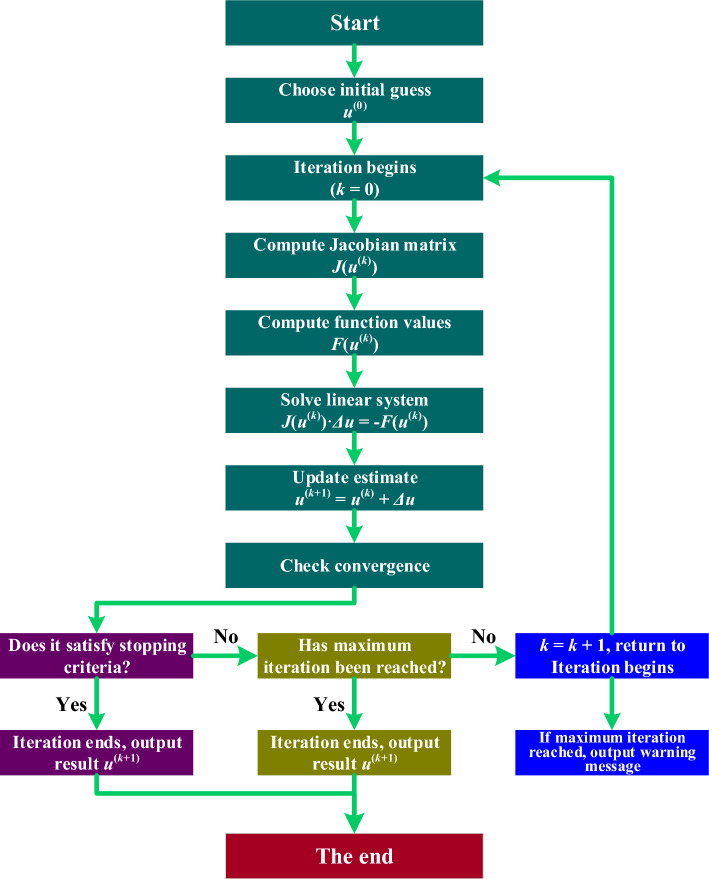
Flowchart Illustrating the Jacobi Matrix Iterative Method.

To tackle this nonlinear equation system, the Jacobi Matrix Iterative Method is employed^47–50^. The detailed methodology for solving this system is outlined as follows^51^:

1. **Initialization**: Set initial estimates for the variables ${u}^{\left(0\right)}=[{m}^{\left(0\right)},{b}_{1}^{\left(0\right)},{b}_{2}^{\left(0\right)}]$.
2. **Iteration Process**:
  - Compute the Jacobian Matrix *J*(*u*^(*k*)^) at each iteration *k*.
  - Compute function values *F*(*u*^(*k*)^).
  - Solve the linear system *J*(*u*^(*k*)^)∙*Δu* = −*F*(*u*^(*k*)^) to find the correction vector *Δu*.
  - Update the estimate *u*^(*k*+1)^ = *u*^(*k*)^ + *Δu*.
  - Check for convergence and repeat until the solution is sufficiently accurate.
  - If termination conditions are not met, restart from step 2a. This ensures continual refinement until desired precision is achieved. Upon meeting the criteria, the iteration concludes, ensuring accuracy and reliability of the numerical solution.
3. **Output**: At the end of the iterative sequence, the resultant estimate, ${u}^{(k+1)}$, stands as the definitive numerical solution for the specified nonlinear equation system. Achieved through meticulous iterative refinement, this outcome represents the convergence of the numerical algorithm toward an accurate approximation of the elusive variables within the system. The significance of this resolution is paramount in scientific research and computational analysis, ensuring precision in handling complex mathematical constructs. A graphical representation, detailing the complete methodology of the Jacobian Matrix Iterative Method, is illustrated in Fig. 8.

In employing the Jacobi Matrix Iterative Method for solving nonlinear equation systems, selecting an appropriate initial estimate for the unknown variables is crucial. This initial guess should closely approximate the actual values, facilitating efficient convergence towards accurate solutions. If the initial estimate is not sufficiently accurate, reaching correct solutions for the nonlinear system becomes markedly more challenging, and may border on impractical. Therefore, the careful choice of an initial estimate is a critical aspect of effectively applying the Jacobi Matrix Iterative Method, vital for attaining precise and significant results.

To address this issue, our research introduces an innovative strategy known as the 'dimensional iteration method'. Initially, we defined fixed dimensions for the FPC, with a total length of $L0$ = 50 mm, coordinate differences at the FPC ends of Δ*x*0 = 20 mm and Δ*y*0 = 0, and initial values of ${m}^{\left(0\right)}$ = -0.00638, ${b}_{1}^{\left(0\right)}$ = 0.11574, and ${b}_{2}^{\left(0\right)}$ = -0.39471. However, in practical scenarios, the actual total length *L*, and the variations in coordinates at the FPC ends, Δ*x* and Δ*y*, frequently diverge from these predetermined values of $L0$, $\Delta x0$, and $\Delta y0$. Consequently, the initially assumed values of ${m}^{\left(0\right)}$, ${b}_{1}^{\left(0\right)}$ and ${b}_{2}^{\left(0\right)}$ might not accurately reflect the true parameters.

In our analytical method, we select a positive integer *N* to discretize the variations in parameters *L*, *m*, *b*_1_ and *b*_2_ into *N* equal segments. This partitioning process is defined as follows:

31 $$dL = {{\left( {L - L0} \right)} \mathord{\left/ {\vphantom {{\left( {L - L0} \right)} N}} \right. \kern-0pt} N}$$

32 $$d\Delta x = {{\left( {\Delta x - \Delta x0} \right)} \mathord{\left/ {\vphantom {{\left( {\Delta x - \Delta x0} \right)} N}} \right. \kern-0pt} N}$$

33 $$d\Delta y = {{\left( {\Delta y - \Delta y0} \right)} \mathord{\left/ {\vphantom {{\left( {\Delta y - \Delta y0} \right)} N}} \right. \kern-0pt} N}$$

The initial FPC curve under consideration is defined by the parameters $L1=L0+dL$, $\Delta x1=\Delta x0+d\Delta x$, and $\Delta y1=\Delta y0+d\Delta y$. By incorporating the initial values ${m}^{\left(0\right)}$, ${b}_{1}^{\left(0\right)}$, and ${b}_{2}^{\left(0\right)}$ with the Jacobi Matrix Iterative Method, we determine the parameters ${m}^{\left(1\right)}$, ${b}_{1}^{\left(1\right)}$, and ${b}_{2}^{\left(1\right)}$ for the first iteration of the FPC curve. Sequentially, parameters for subsequent FPC curves (such as ${m}^{\left(2\right)}$, ${b}_{1}^{\left(2\right)}$, and ${b}_{2}^{\left(2\right)}$ for the second iteration, and so on) are systematically derived based on the previously computed values. This iterative process continues until the slope ${m}^{\left(N\right)}$ and intercepts ${b}_{1}^{\left(N\right)}$ and ${b}_{2}^{\left(N\right)}$ for the *N*-th FPC curve iteration are accurately identified.

Using the calculated values of the slope '*m*' and intercepts '*b*_1_' and '*b*_2_', the coordinates of any specific point along the FPC can be precisely determined according to Eqs. (34) and (35):

34 $$x = x_{0} + \int_{0}^{l} {\cos \left( {\theta \left( l \right)} \right)} dl$$

35 $$y = y_{0} + \int_{0}^{l} {\sin \left( {\theta \left( l \right)} \right)} dl$$

In these equations, the notation (${x}_{0}$, ${y}_{0}$) represents the coordinates of the initial point of the FPC, while (*x*, *y*) denotes the coordinates of any specific point on the FPC. The variable *θ* signifies the tangent angle at any point along the circuit. If the point in question is situated within Segment *S*1, its mathematical representation conforms to Eq. (13). Alternatively, if the point is located in Segment *S*2, its expression is governed by Eq. (20).

**Figure 9:**
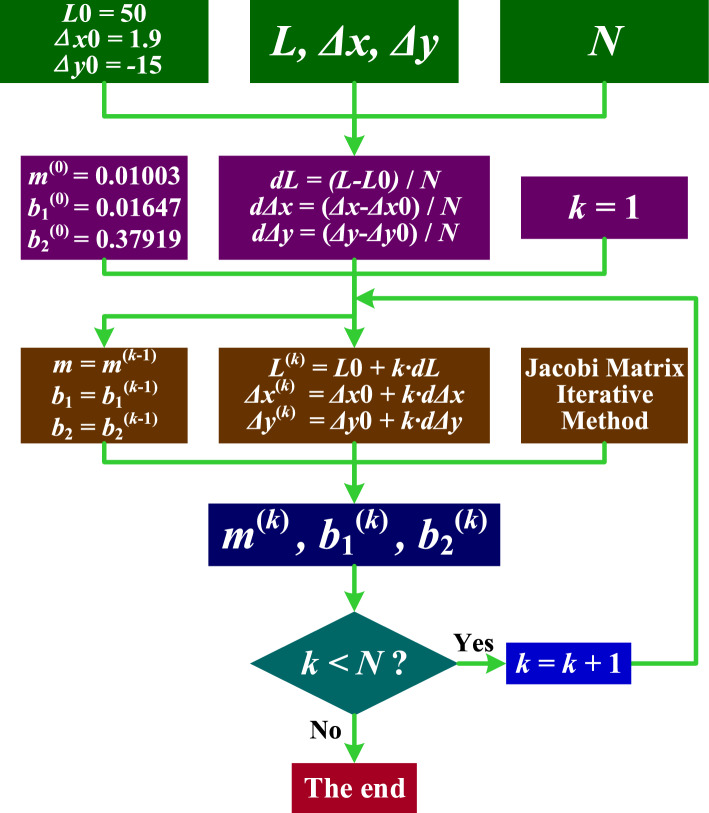
The flowchart of the dimensional iteration method.

The final curve, identified as the *N*-th curve, represents the actual FPC curve necessitating comprehensive analysis. This extensive analytical procedure is visually delineated and explained in the flowchart depicted In Fig. 9.

When the values of '*m*', '*b*_1_' and '*b*_2_' prove elusive, marked by significant discrepancies between their estimated and actual values in earlier iterations, an efficacious strategy is to increase the value of *N*. Generally, a threshold of 50 is deemed adequate for the precise computation of these parameters in most cases, thus enhancing the validity and reliability of our results.

### Iterative analysis and comparative results

**Figure 10:**
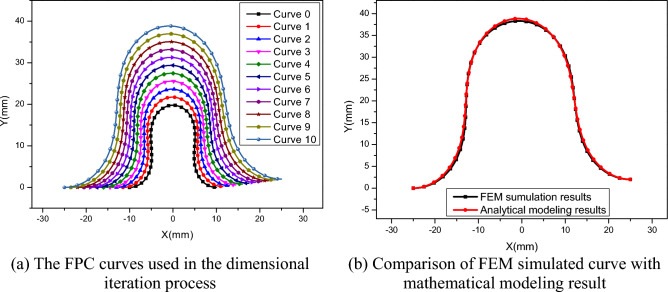
Dimensional Iteration Process and Comparative Evaluation of FEM Simulation Versus Mathematical Modeling.

In this study, we set *N* to 10, meaning that the computational process underwent ten iterations, as illustrated In Fig. 10a. Curve 0 outlines the stable FPC configuration and serves as the starting point for the iterative process. Following each iteration, a new FPC curve emerges, each characterized by distinct dimensions and overall length. Notably, Curve 10, resulting from the tenth iteration, signifies the critical outcome of our analysis. The entire iterative series proceeded smoothly, underscoring the methodological robustness and reliability of our approach.

Figure 10b displays a comparative analysis between the curve simulated using the Finite Element Method (FEM) and the curve resulting from analytical modeling.

## Design optimization strategies

### Free-contact optimization

**Figure 11:**
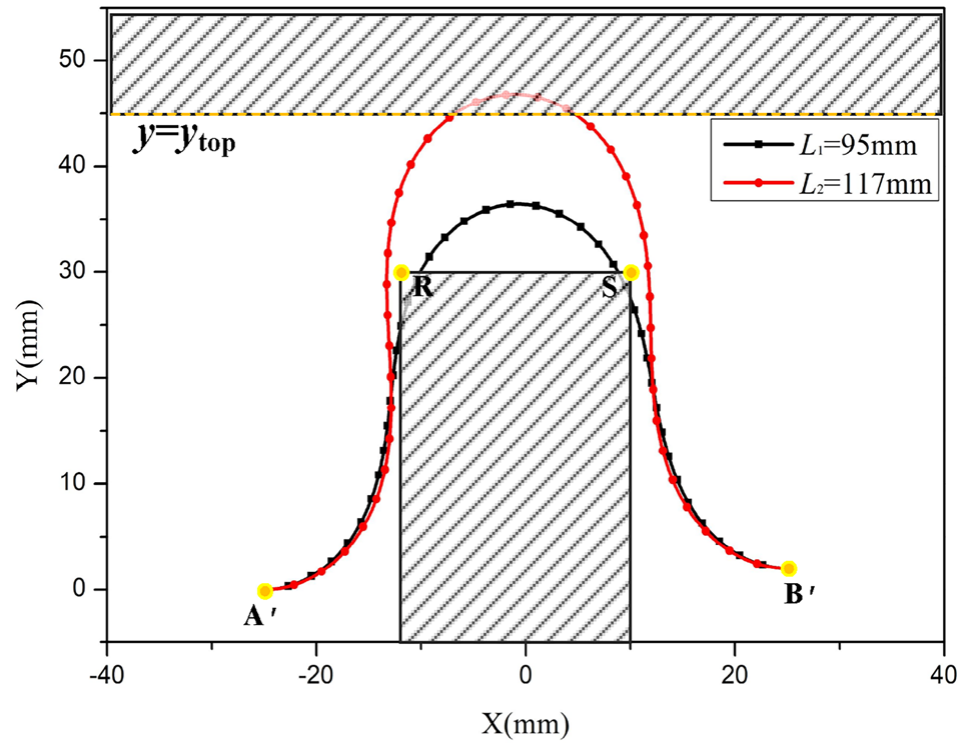
Curves with Inappropriate Lengths Resulting in Contact with Adjacent Components.

Figure 11 illustrates the areas shaded in green, which represent components adjacent to the FPC. The FPCs with lengths of 100 mm and 117 mm demonstrate interactions with different components. To ensure the FPC avoids any contact with these adjacent components, optimizing its length is essential.

The coordinates of Point R are denoted as (*x*_R_, *y*_R_), and those of Point S as (*x*_S_, *y*_S_), where *y*_R_ equals *y*_S_. The y-coordinate of the upper component is represented as ytop. For any point M on the FPC, characterized by coordinates (*x*_M_, *y*_M_), if its coordinates satisfy either of the following conditions:

1. *y*_M_ > *y*_top_,
2. *x*_R_ < *x*_M_ < *x*_S_ and *y*_M_ < *y*_S_,

then it can be concluded that the FPC is interacting with another component.

To determine the suitable range of lengths for the FPC, the iterative incrementation method was employed. This approach involves progressively increasing the FPC's length in steps. Following each increment, the curve of the FPC was analyzed to ascertain whether any point on the FPC meets the previously specified conditions.

The initial value of the FPC length is set as the Euclidean distance between points A’ and B’. However, in most cases, this FPC length renders Eq. (30) unsolvable. Therefore, the length should be incrementally increased until Eq. (30) can be accurately solved.

To optimize time efficiency and enhance accuracy, the large step size and small step size were designated as Δ*L* = 5 mm and δ*L* = 0.1 mm, respectively.

**Figure 12:**
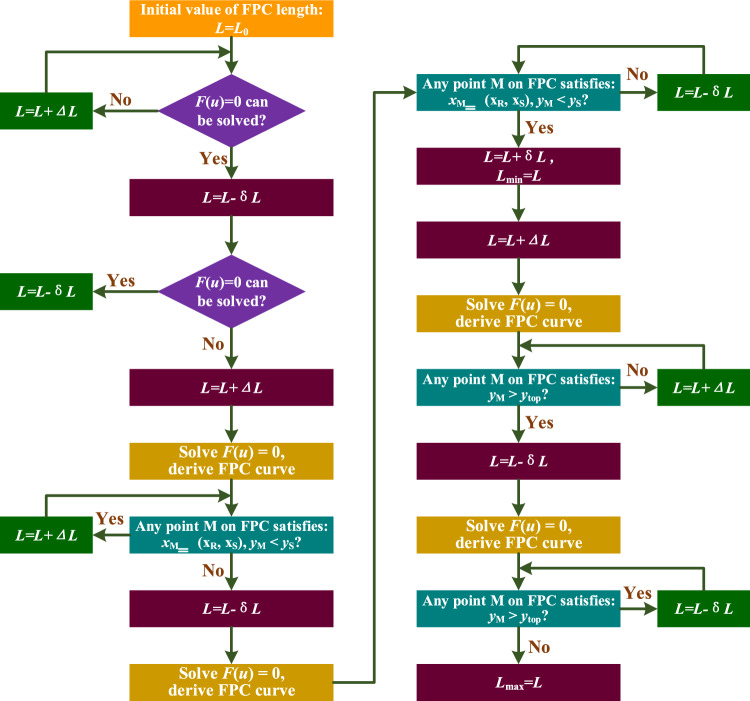
Flowchart Illustrating the Procedure to Determine the Optimal FPC Length Range for Free Contact.

The flowchart depicting the process for determining the appropriate range of the FPC length is illustrated In Fig. 12.

**Figure 13:**
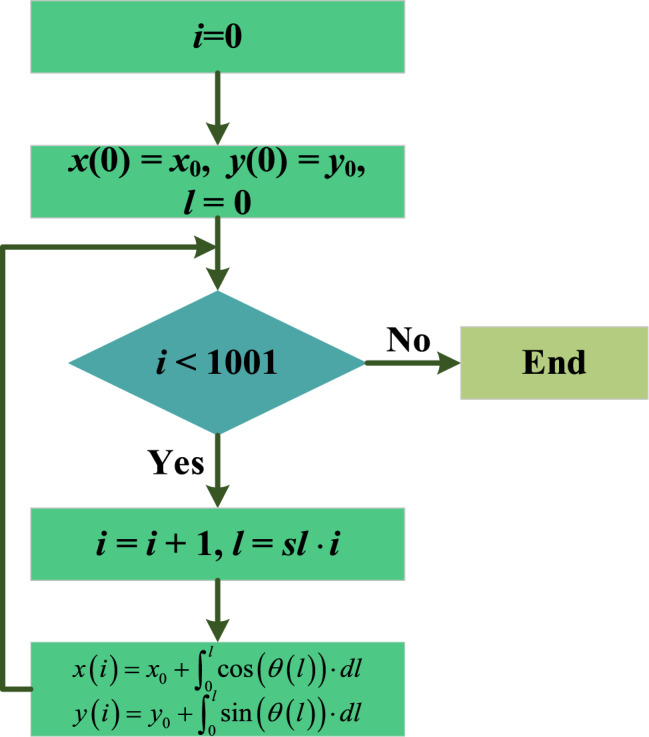
Flowchart Depicting the Methodology for Identifying a Series of Points and Their Corresponding Coordinates on the Deformed FPC.

To determine whether any point on FPC resides within the specified range, the FPC's length was systematically divided into 1000 equal segments, with each segment measuring *sl* = *L*/1000 in length. This segmentation yields 1001 points along the FPC. For each point, it is essential to ascertain its corresponding coordinate. The procedure for identifying these points and their associated coordinates is outlined in the flowchart depicted In Fig. 13.

**Figure 14:**
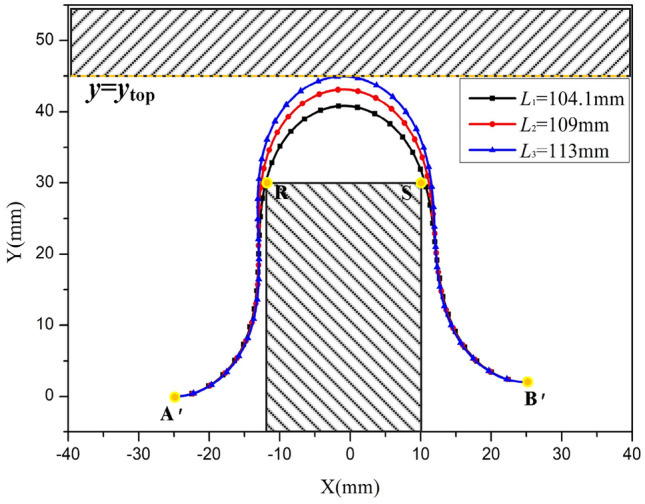
Bell-Shaped FPC Curves Within the Optimized Length Range.

In alignment with the previously delineated theoretical framework, the permissible range for the length of the FPC is defined as: 104.1 mm ≤ *L* ≤ 113 mm. The FPC curves, with respective lengths of *L*_1_ = 104.1 mm, *L*_2_ = 109 mm, and *L*_3_ = 113 mm, are depicted In Fig. 14. Examination reveals that all these curves successfully avoid contact with adjacent components, with the curve having a length of *L*_2_ = 109 mm being particularly noteworthy in this regard.

### Optimization of curvature

The meticulous regulation of the curvature in FPCs is imperative to mitigate a spectrum of detrimental consequences. Excessive curvature of FPCs can precipitate a multitude of complications, such as mechanical stress within the circuit, which may culminate in intermittent or permanent impairment of the circuit's functionality. Additionally, suboptimal heat dissipation due to high curvature can abbreviate the circuit's lifespan and compromise the device's reliability. Further, pronounced curvature heightens the FPC's susceptibility to physical damage, both during assembly and throughout its operational tenure. From a manufacturing standpoint, sustaining moderate curvature in FPCs is crucial to facilitate assembly processes and ensure compatibility with other electronic components^52^.

Therefore, it is imperative to design and manufacture FPCs with an optimal curvature that balances flexibility with structural integrity and functional reliability.

**Figure 15:**
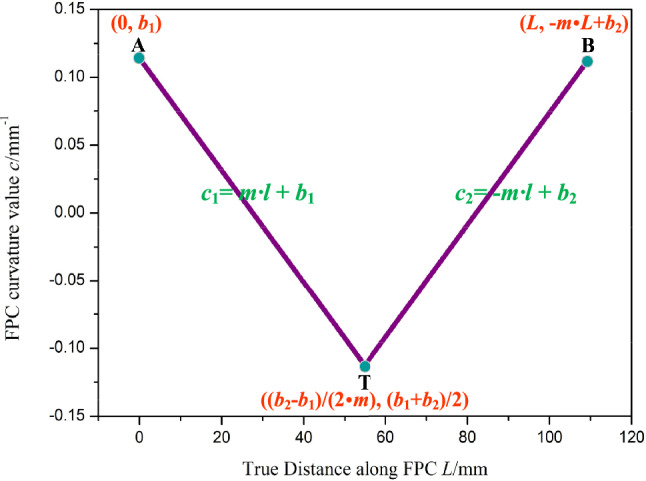
Ideal Curvature Trend of the FPC as Utilized in the Analytical Model.

The observed curvature trend of the FPC, as derived from the finite element model, is depicted In Fig. 6, whereas the ideal curvature trend employed in the analytical model is illustrated In Fig. 15. Points A and B designate the commencement and termination of the FPC's curvature, respectively, while Point T denotes the location on the FPC where the curvature begins to alter. It is deducible that the apex of the curvature value is confined to the region demarcated by these three points.

The curvature trends of the FPC are characterized by the linear equations *c*_1_ = *m·l* + *b*_1_ and *c*_2_ =  − *m·l* + *b*_2_. The coordinates of Points A, B, and T are defined as (0, *b*_1_), (*L*, − *m·l* + *b*_2_), and ($\frac{{b}_{2}-{b}_{1}}{2\cdot m}, \frac{{b}_{1}+{b}_{2}}{2})$, respectively. Under various specified conditions for the FPC, provided Eq. (30) is resolved, the precise coordinate values for Points A, B, and T can be accurately determined.

**Figure 16:**
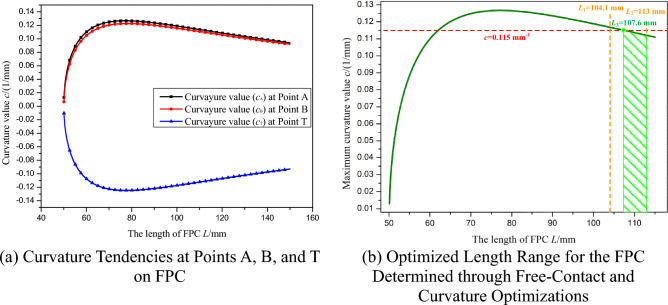
Optimization of FPC Length Based on Curvature Limit for Bell-Shaped Curve.

As the length of the FPC extends from 50.1 mm to 120 mm, the corresponding curvature tendencies at points A, B, and T are illustrated In Fig. 16a.

It is observed that with the initial increase in FPC length, the curvature values at points A, B, and T rise rapidly, reaching their peak values at approximately 70 mm. Beyond this length, the curvature values at these points begin to decrease gradually.

For the maximum curvature *c*_max_, which is defined as:

36 $$c_{{{\text{max}}}} = {\text{max }}\left( {\left| {c_{{\text{A}}} } \right|, \, \left| {c_{{\text{B}}} } \right|, \, |c_{{\text{T}}} |} \right)$$

The trend of the *c*_max_ value is depicted In Fig. 16b. The established curvature requirement stipulates that the curvature at any point on the FPC must not exceed 0.115 mm^-1^. Consequently, the permissible range of FPC length was determined to be between 107.6 mm and 113 mm. This range is indicated by the green-shaded area In Fig. 16b.

The optimal value for the FPC length, denoted as *L*_best_, is defined such that it corresponds to the smallest maximum curvature within the determined range. In this case, *L*_best_ is determined to be 113 mm, a value that can be readily identified In Fig. 16b.

**Figure 17:**
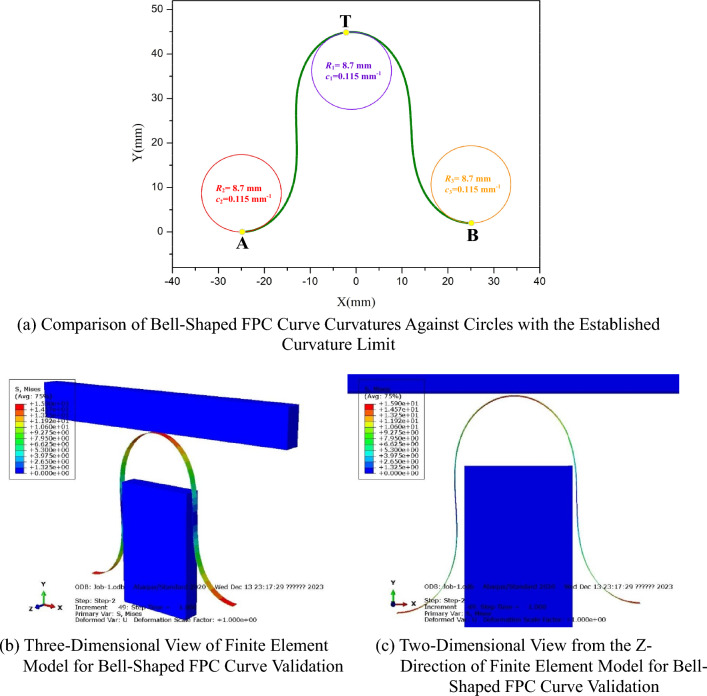
Validation of Curvature and Free-Contact for the Optimal Length Bell-Shaped FPC Curve.

The curve of the FPC, with length value of *L* = 113 mm, is illustrated In Fig. 17a. For comparative analysis, three circles, each with a radius of R = 8.7 mm (yielding a curvature value of *c* = 1/*R* = 0.115 mm^-1^), were superimposed at points A, B, and T. Furthermore, a corresponding finite element simulation was performed, with its results depicted in Fig. 17b,c.

The curvature values of the FPC at points A, B, and T are observed to be less than the established curvature limit of *c* = 0.115 mm^-1^. This indicates that the maximum curvature of the FPC, when optimized in length, meets the curvature requirements, thereby validating the practical reliability of this optimization. Furthermore, finite element simulation results reveal that the FPC curve, with its optimal length, effectively avoids any contact with adjacent components. This evidence collectively substantiates the acceptability of the FPC curve with the optimized length.

## Analysis of two additional conventional FPC curves

### ʹUʹ-shaped FPC curve

**Figure 18:**
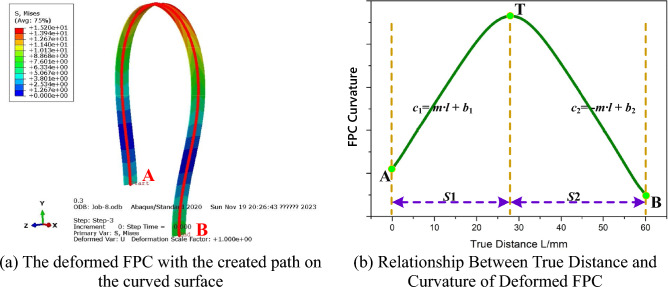
Investigation of Curvature Variation Trends in 'U'-Shaped FPC Curves, Derived from Bending Stresses in the Finite Element Model.

Regarding the 'U'-shaped FPC curve depicted In Fig. 18a, a comparable methodology can be employed for analysis and prediction of its configuration. A trajectory comprising a series of distinct points was meticulously constructed along the trajectory of the FPC curve. By utilizing a similar approach, the inclination of the FPC curvature was successfully determined, as illustrated In Fig. 18b.

To analyze the 'U' shape of the FPC curve, the relevant system of equations is as follows:

37 $$F\left( u \right) = \left[ \begin{gathered} F1 \hfill \\ F2 \hfill \\ F3 \hfill \\ \end{gathered} \right] = \left[ \begin{gathered} \Delta x - \left[ {\int_{0}^{{\frac{{b_{2} - b_{1} }}{2m}}} {\cos \left( {{\pi \mathord{\left/ {\vphantom {\pi 2}} \right. \kern-0pt} 2} - \int_{0}^{l} {\left( {ml + b_{1} } \right)dl} } \right)dl + \int_{{\frac{{b_{2} - b_{1} }}{2m}}}^{L} {\cos \left( {{\pi \mathord{\left/ {\vphantom {\pi 2}} \right. \kern-0pt} 2} - \int_{0}^{{\frac{{b_{2} - b_{1} }}{2m}}} {\left( {ml + b_{1} } \right)dl - \int_{{\frac{{b_{2} - b_{1} }}{2m}}}^{l} {\left( { - ml + b_{2} } \right)dl} } } \right)dl} } } \right] \hfill \\ \Delta y - \left[ {\int_{0}^{{\frac{{b_{2} - b_{1} }}{2m}}} {\sin \left( {{\pi \mathord{\left/ {\vphantom {\pi 2}} \right. \kern-0pt} 2} - \int_{0}^{l} {\left( {ml + b_{1} } \right)dl} } \right)dl + \int_{{\frac{{b_{2} - b_{1} }}{2m}}}^{L} {\sin \left( {{\pi \mathord{\left/ {\vphantom {\pi 2}} \right. \kern-0pt} 2} - \int_{0}^{{\frac{{b_{2} - b_{1} }}{2m}}} {\left( {ml + b_{1} } \right)dl - \int_{{\frac{{b_{2} - b_{1} }}{2m}}}^{l} {\left( { - ml + b_{2} } \right)dl} } } \right)dl} } } \right] \hfill \\ \Delta \theta - \left[ { - \int_{0}^{{\frac{{b_{2} - b_{1} }}{2m}}} {\left( {ml + b_{1} } \right)dl - \int_{{\frac{{b_{2} - b_{1} }}{2m}}}^{L} {\left( { - ml + b_{2} } \right)dl} } } \right] \hfill \\ \end{gathered} \right] = 0$$

The distinction between Eq. (37), which delineates a 'U'-shaped curve, and Eq. (30), characterizing a bell-shaped curve, lies in their respective tangent angles at the commencement and termination points. Specifically, for the 'U'-shaped curve, the tangent angle initiates at π/2 and concludes at -π/2. In contrast, the bell-shaped curve starts and ends with a tangent angle of 0. Consequently, the variations in tangent angles from the starting to the ending points are -π for the 'U'-shaped curve and 0 for the bell-shaped curve.

For the adjacent components delineated by the points P, Q, R, and S, with coordinates (-9.5, 29), (10.5, 29), (-7, 20), and (7, 20) respectively, a designated area within this geometric configuration is allocated for the FPC. The FPC extends from its starting point A', situated at (-7.05, 0), to its end point B', located at (-7.05, -2).

In accordance with the theoretical framework analogous to that depicted In Fig. 12, the optimal range for the FPC length was established to be 62.4 mm ≤ *L* ≤ 70.3 mm. The suitability of the FPC curve is adjudicated based on whether any given point Pi (*x*_i_, *y*_i_) on the FPC fulfills at least one of the ensuing criteria:

1. *x*_R_ < *x*_i_ < *x*_S_ and *y*_i_ < *y*_R_;
2. *x*_i_ < *x*_P_;
3. *x*_i_ > *x*_Q_;
4. *y*_i_ > *y*_P_.

**Figure 19:**
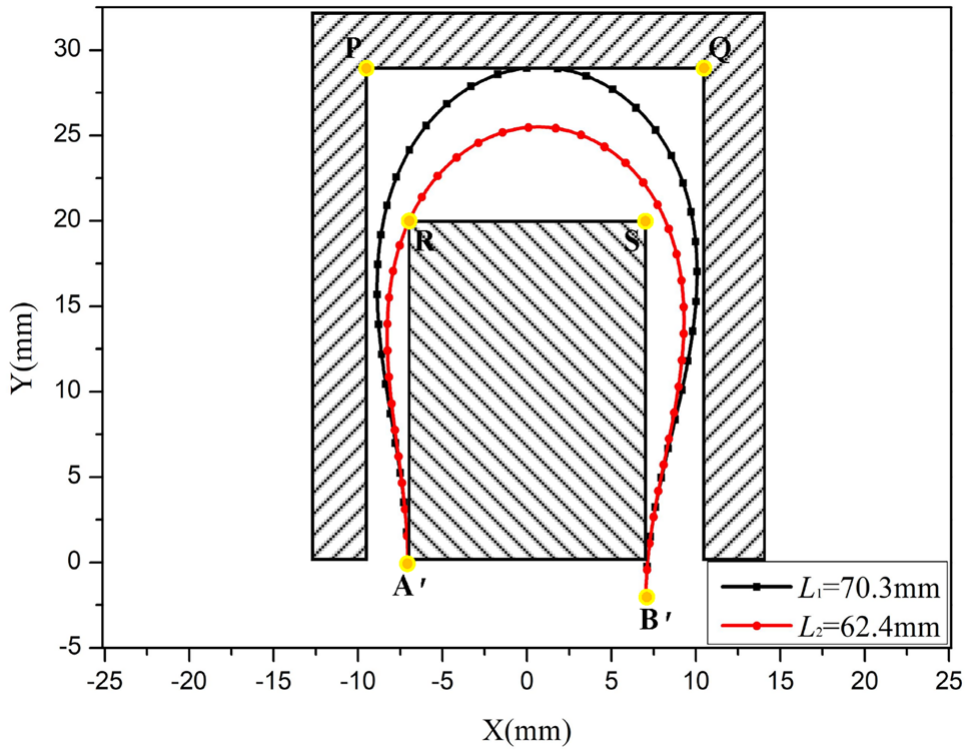
Illustration of 'U'-Shaped FPC Curves within the Optimally Determined Length Range, Alongside Adjacent Components.

The FPC curves corresponding to lengths *L*_1_ = 70.3 mm and *L*_2_ = 62.4 mm are depicted In Fig. 19.

**Figure 20:**
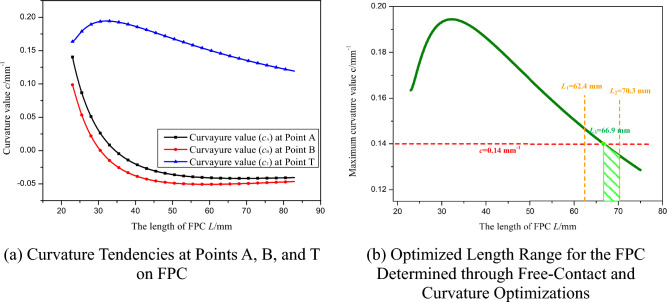
Length Optimization of FPC Considering the Curvature Constraints for 'U'-Shaped Configuration.

The curvature tendencies at points A, B, and T are depicted In Fig. 20a. For the optimization of the FPC curvature, emphasis was given to the curvature values exhibiting the largest absolute values. This approach aligns with the curvature tendency at Point T, as illustrated In Fig. 20b.

As delineated In Fig. 20b, the FPC length range, demarcated by two dashed yellow lines, was determined through an optimization process aimed at preventing contact with adjacent components. The curvature limit set for this optimization was *c* = 0.14 mm^-1^, represented by a red dashed line In Fig. 20b. Consequently, this establishes the appropriate FPC length range as 66.9 mm ≤ *L* ≤ 70.3 mm. The optimal FPC length within this suitable range, which yields the curve with the smallest absolute curvature value, is identified as the most advantageous length for the FPC, designated in this instance as *L*_3_ = 70.3 mm.

**Figure 21:**
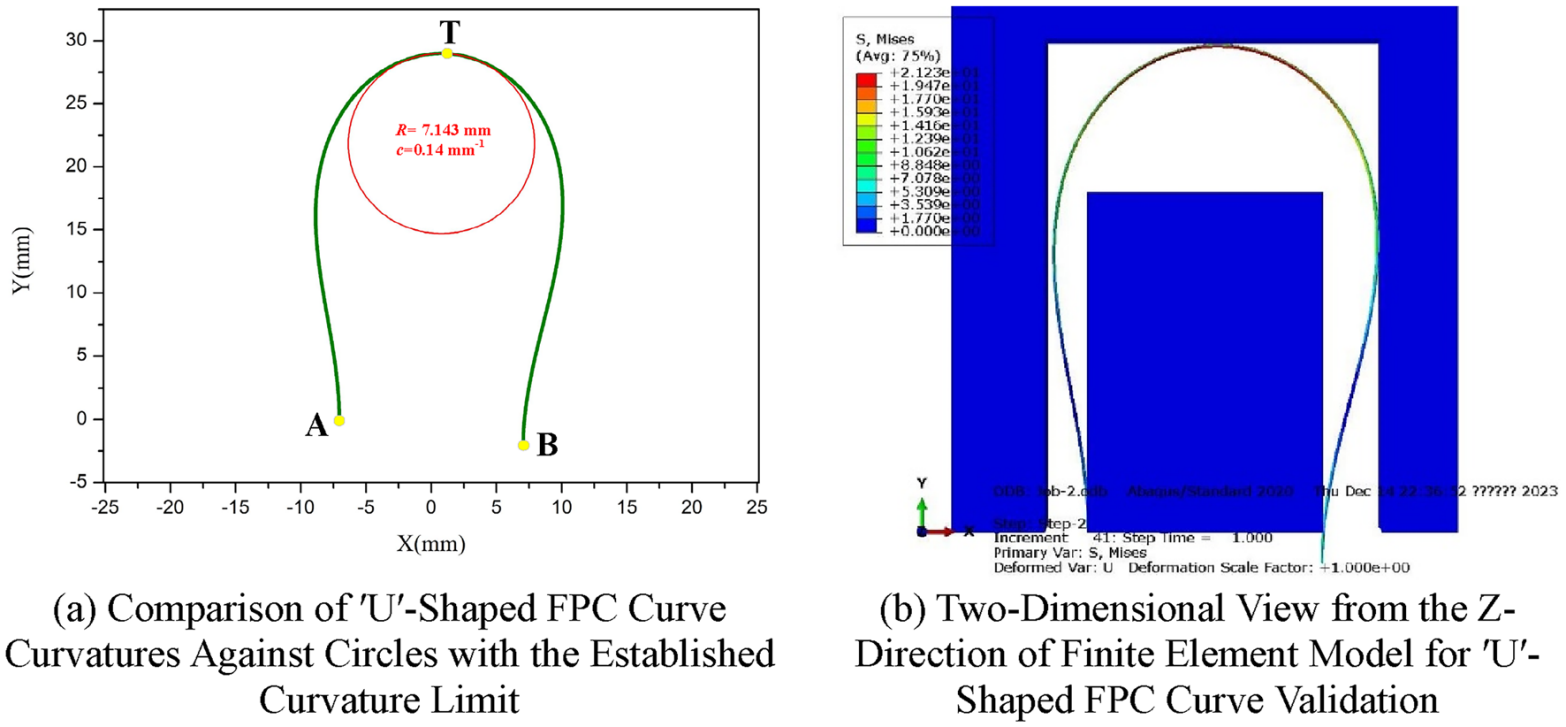
Verification of Curvature Compliance and Non-Contact Conditions in the Optimally Lengthened 'U'-Shaped FPC Curve.

The FPC curve, with a length of *L* = 70.3 mm, is depicted In Fig. 21a. For comparative purposes, a circle possessing a radius of *R* = 7.143 mm (equating to a curvature value of *c* = 1/*R* = 0.14 mm^-1^) was superimposed at point T. Additionally, a corresponding finite element simulation was conducted, the results of which are presented In Fig. 21b.

Observations reveal that the curvature of the FPC at point T is below the established curvature limit of 0.14 mm^-1^. Moreover, the FPC curve adeptly avoids contact with adjacent components. These factors collectively indicate that the optimized FPC length, as determined through the aforementioned process, is both effective and practical.

### ʹSʹ-shaped FPC curve

**Figure 22:**
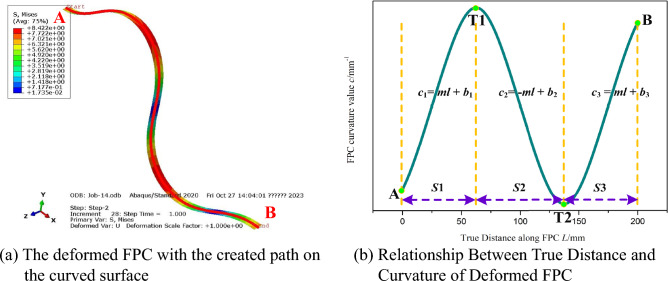
Investigation of Curvature Variation Trends in 'S'-Shaped FPC Curves, Derived from Bending Stresses in the Finite Element Model.

Regarding the 'S'-shaped curve of the FPC, the finite element model incorporating the created path, as well as the relationship between the actual distance along the FPC and the curvature of the deformed FPC, are respectively depicted in Fig. 22a,b.

Contrasting with the previously presented bell-shaped and 'U'-shaped FPC curves, the 'S'-shaped curve of the FPC in this instance necessitates segmenting the relationship between true distance and curvature of deformed FPC into three distinct parts, as illustrated in Fig. 22b. Since the 'S'-shaped curve exhibits rotational symmetry, the relationship between the true distance and curvature of the deformed FPC, as depicted In Fig. 22b, must intersect the point (*L*/2, 0). This necessitates the condition $-m\cdot l+{b}_{2}=0$ to be satisfied. To analyze the 'S'-shaped curve of the FPC, the corresponding system of equations is presented as follows:

38 $$F\left( u \right) = \left[ \begin{gathered} F1 \hfill \\ F2 \hfill \\ F3 \hfill \\ F4 \hfill \\ \end{gathered} \right] = \left[ \begin{gathered} \Delta x - \left( {\int_{0}^{{\left( {b_{2} - b_{1} } \right)/2m}} {\cos \left( {\theta_{S1} } \right)dl + \int_{{\left( {b_{2} - b_{1} } \right)/2m}}^{{\left( {b_{2} - b_{3} } \right)/2m}} {\cos \left( {\theta_{S2} } \right)dl + \int_{{\left( {b_{2} - b_{3} } \right)/2m}}^{L} {\cos (\theta_{S3} )dl} } } } \right) \hfill \\ \Delta y - \left( {\int_{0}^{{\left( {b_{2} - b_{1} } \right)/2m}} {\sin \left( {\theta_{S1} } \right)dl + \int_{{\left( {b_{2} - b_{1} } \right)/2m}}^{{\left( {b_{2} - b_{3} } \right)/2m}} {\sin \left( {\theta_{S2} } \right)dl + \int_{{\left( {b_{2} - b_{3} } \right)/2m}}^{L} {\sin (\theta_{S3} )dl} } } } \right) \hfill \\ \Delta \theta - \left( { - \int_{0}^{{\left( {b_{2} - b_{1} } \right)/2m}} {\left( {ml + b1} \right)dl - \int_{{\left( {b_{2} - b_{1} } \right)/2m}}^{{\left( {b_{2} - b_{3} } \right)/2m}} {\left( { - ml + b2} \right)dl - \int_{{\left( {b_{2} - b_{3} } \right)/2m}}^{L} {(ml + b3)dl} } } } \right) \hfill \\ - mL/2 + b_{2} \hfill \\ \end{gathered} \right] = 0$$

where ${\theta }_{S1}$, ${\theta }_{S2}$ and ${\theta }_{S3}$ are the tangent angles within the *S*1, *S*2, and *S*3 segments, respectively. These angles can be calculated as follows:

39 $$\theta_{S1} = - \int_{0}^{l} {\left( {ml + b_{1} } \right)dl}$$

40 $$\theta_{S2} = \theta_{T1} - \int_{{\left( {b_{2} - b_{1} } \right)/2m}}^{l} {\left( { - ml + b_{2} } \right)dl}$$

41 $$\theta_{S3} = \theta_{T2} - \int_{{\left( {b_{2} - b_{3} } \right)/2m}}^{l} {\left( {ml + b_{3} } \right)dl}$$

Here, ${\theta }_{T1}$ and ${\theta }_{T2}$ are the tangent angles at points T1 and T2, respectively, and are expressed as:

42 $$\theta_{T1} = - \int_{0}^{{\left( {b2 - b1} \right)/2m}} {\left( {ml + b_{1} } \right)dl}$$

43 $$\theta_{T2} = - \int_{0}^{{\left( {b_{2} - b_{1} } \right)/2m}} {\left( {ml + b_{1} } \right)dl} - \int_{{\left( {b_{2} - b_{1} } \right)/2m}}^{{\left( {b_{2} - b_{3} } \right)/2m}} {\left( { - ml + b_{2} } \right)dl}$$

The 'S'-shaped curve initiates and terminates at a tangent angle of 0. As a result, the variation in tangent angles from the starting point to the ending point, denoted as Δ*θ*, is 0.

For the adjacent components identified by points R and S, with coordinates (-4, 18) and (4, -18) respectively, a confined space is allocated for the FPC. The FPC originates at point A', positioned at (-20, 30), and terminates at point B', located at (20, -30), as depicted In Fig. 24.

**Figure 23:**
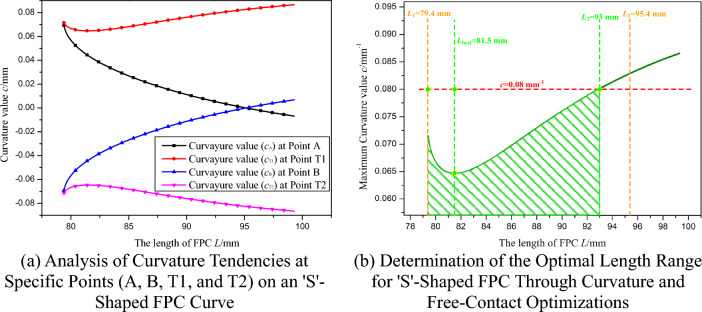
Length Optimization of FPC Considering Curvature Constraints in 'S'-Shaped Configuration.

Aligned with the theoretical model similar to that illustrated In Fig. 12, the ideal length range for the FPC has been determined to be between 79.4 mm and 95.4 mm, which are represented by two dashed yellow lines In Fig. 23b. The appropriateness of the FPC curve is assessed by examining if any specific point Pi (*x*_i_, *y*_i_) on the FPC meets at least one of the following conditions:

1. *x*_i_ < *x*_R_ and *y*_i_ < *y*_R_;
2. *x*_i_ > *x*_S_ and *y*_i_ > *y*_S_;

The curvature tendencies of the FPC at points A, B, T1, and T2, as the length of the FPC increases, are illustrated in Fig. 23a. For the optimization of FPC curvature, the curvature values exhibiting the highest absolute magnitude were prioritized, corresponding to the curvature pattern at point T1. The established curvature threshold for this optimization is *c* = 0.08 mm^-1^, denoted by a red dashed line in Fig. 23b.

The previously determined permissible length range for the FPC, ensuring the avoidance of contact with adjacent components, is defined as 79.4 mm ≤ L ≤ 95.4 mm. Within this range, a more specific interval of 79.4 mm ≤ L ≤ 93 mm is established, aligning the FPC's curvature within the prescribed threshold. This interval corresponds to the region shaded in green in Fig. 23b. Significantly, the FPC attains its minimal curvature at a length of 93 mm, delineating this measurement as the optimal length.

**Figure 24:**
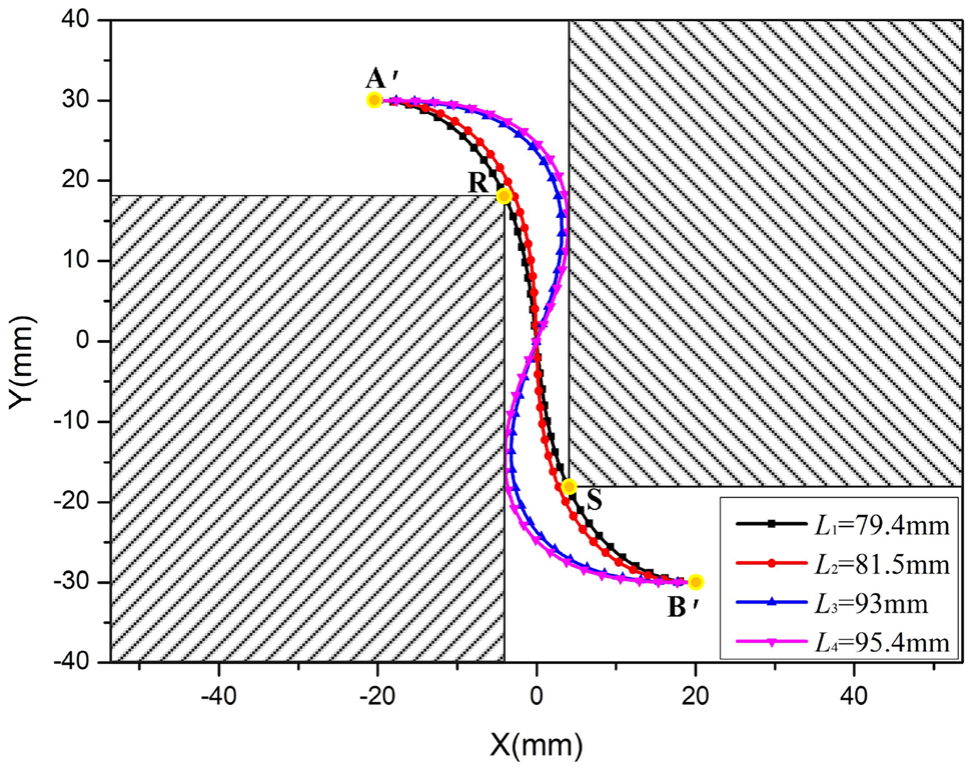
Illustration of 'S'-Shaped FPC Curves within the Optimally Determined Length Range, Alongside Adjacent Components.

The trajectories of the FPC, with respective lengths of *L*_1_ = 79.4 mm, *L*_2_ = 81.5 mm, *L*_3_ = 93 mm, and *L*_4_ = 95.4 mm, are illustrated in Fig. 24. Notably, each curve is strategically designed to avoid any contact with the adjacent components, which are demarcated by the area shaded in black.

**Figure 25:**
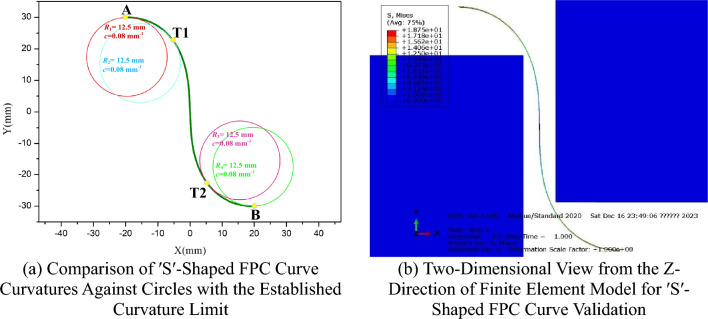
Verification of Curvature Compliance and Non-Contact Conditions in the Optimally Lengthened 'S'-Shaped FPC Curve.

For the FPC curve with a length of *L* = 81.5 mm, identified as the optimal length in this context, the validation of its curvature is depicted in Fig. 25a. Here, four circles, each with a radius of *R* = 12.5 mm (corresponding to a curvature limit value of *c* = 1/*R* = 0.08 mm^-1^), are superimposed at points A, B, T1, and T2. Furthermore, Fig. 25b illustrates the validation of the curve's free-contact status with adjacent components, as analyzed through finite element modeling.

It is evident from the analysis that the curvature values of the FPC at the four designated points conform to the prescribed curvature limit. Additionally, the curve of the FPC successfully avoids any contact with adjacent components. This observation substantiates the reliability and practicality of the optimized S-shaped curve in this application.

## Conclusions

This investigation conducts an exhaustive analysis of FPCs, focusing on optimizing their design to achieve two primary objectives: averting contact with adjacent components and adhering to specified curvature parameters. Utilizing Finite Element Method (FEM) simulations in conjunction with the Jacobi Matrix Iterative Method, we refined mathematical modeling techniques to establish a predictive model characterizing the deformation behavior of FPCs under a variety of conditions. Our research employs an advanced optimization process to examine various FPC configurations, including bell, 'U', and 'S' shapes, emphasizing the importance of spatial placement to prevent mechanical interference and managing curvature to mitigate mechanical stress, thereby preempting circuit failure. This process facilitates the development of FPCs that seamlessly integrate into their designated spatial confines while upholding structural integrity and reliable functionality throughout their operational lifespan.

Our research introduces a cutting-edge approach to mathematical modeling, which significantly enhances the accuracy and applicability of our predictive models for FPC behavior. The innovative application of the Jacobi Matrix Iterative Method further refines these models, enabling precise adjustments and higher fidelity in simulation results compared to traditional methods. These methodological advancements not only contribute to our understanding of FPC dynamics but also enhance the practical outcomes in FPC design and application.

The findings from this research underscore the critical importance of managing FPC curvature and strategic placement to prevent contact with neighboring components. This dual approach is pivotal in diminishing mechanical stress and enhancing the longevity of FPCs, particularly in scenarios demanding high flexibility and precision. The implications of our innovative modeling techniques extend significantly across various industries, such as aerospace, medical technology, automotive, and consumer electronics. They offer crucial insights for optimizing FPC design, overcoming curvature limitations, and preventing physical interference with adjacent components, thus augmenting both performance and durability.

In conclusion, this research provides a comprehensive perspective on the design and implementation of FPCs, underscoring the necessity of managing both physical space and mechanical properties. The methodological innovations introduced offer a robust framework for advancing FPC technology, ensuring these vital components meet the evolving demands of modern electronics.

## Data Availability

The datasets generated and analyzed during the current study are not publicly available due to confidentiality agreements and company policies regarding proprietary data. However, the data that support the findings of this study are available from the corresponding author upon reasonable request and with the approval of Nanjing WIT Science & Technology Co., Ltd.. Correspondence and requests for materials should be addressed to Yicai Shan at danyicai@njxzc.edu.cn.
